# Perceived differences in social status between speaker and listener affect the speaker's vocal characteristics

**DOI:** 10.1371/journal.pone.0179407

**Published:** 2017-06-14

**Authors:** Juan David Leongómez, Viktoria R. Mileva, Anthony C. Little, S. Craig Roberts

**Affiliations:** Faculty of Natural Sciences, University of Stirling, Stirling, United Kingdom; Macquarie University, AUSTRALIA

## Abstract

Non-verbal behaviours, including voice characteristics during speech, are an important way to communicate social status. Research suggests that individuals can obtain high social status through dominance (using force and intimidation) or through prestige (by being knowledgeable and skilful). However, little is known regarding differences in the vocal behaviour of men and women in response to dominant and prestigious individuals. Here, we tested within-subject differences in vocal parameters of interviewees during simulated job interviews with dominant, prestigious, and neutral employers (targets), while responding to questions which were classified as introductory, personal, and interpersonal. We found that vocal modulations were apparent between responses to the neutral and high-status targets, with participants, especially those who perceived themselves as low in dominance, increasing fundamental frequency (F_0_) in response to the dominant and prestigious targets relative to the neutral target. Self-perceived prestige, however, was less related to contextual vocal modulations than self-perceived dominance. Finally, we found that differences in the context of the interview questions participants were asked to respond to (introductory, personal, interpersonal), also affected their vocal parameters, being more prominent in responses to personal and interpersonal questions. Overall, our results suggest that people adjust their vocal parameters according to the perceived social status of the listener as well as their own self-perceived social status.

## Introduction

In hierarchical social relationships, individuals who are of high social status normally have privileges that other members of their group lack [1]. Examples of this type of relationship in human societies include the ranking system within the military and company organisation models (e.g. an employer is higher in social status than an employee) [1]. Recent research suggests that individuals can obtain high social status through one of two main ways: by using force and intimidation (dominance), or by being knowledgeable and skilful (prestige) [2,3]. Humans communicate their social status to others using a wide range of behaviours, often shared with non-human animals, such as facial expressions and body postures [4], linguistic cues, like the use of formal and informal linguistic tenses, as well as using spatial metaphors that make reference to hierarchies or imply a large personal space [1,5].

In terms of non-verbal behaviour, alongside facial expressions and body postures, voice characteristics are an important means to communicate socially relevant information, including social status (e.g. [6,7,8]). The acoustic qualities of the human voice, aside from linguistic elements such as syntax and semantic content, can communicate an important array of biological information about the speaker including sex, femininity, attractiveness, fertility and sexual maturity, physical strength, and body size [9–23]. Human voices are sexually dimorphic, with men, for example, having lower pitched voices than women. While the precise evolutionary reasons for this pronounced difference are unclear, it has been suggested that it could be a product of sexual selection [24], including dominance competition [25]. In fact, there is strong cross-species evidence for the influence of sexual selection on male fundamental frequency (F_0_), the parameter most closely related to voice pitch [26].

While no research to our knowledge has explored vocal parameters with respect to prestige, the effects of dominance have been widely studied. In general, studies have found that voices low in F_0_ are perceived as more dominant in both men [6,7] and women [8]; however, one study [27] found a significant positive correlation between F_0_ and dominance judgments for male, but not female, speakers. This discrepancy can be due to several reasons: first, they used vocal recordings that were approximately 3.5 seconds in length, which may be too short to base dominance judgements on. Additionally, the authors’ paradigm was very complex with several different contexts, which they suggest may affect the findings. Finally, the authors used an unspecified pitch contour to calculate their mean vocal parameters, and thus their calculations of pitch may have differed from others.

Perceptions of dominance appear to be based on multiple cues: F_0_, which is related to androgen levels, as well as formant dispersion (D_f_), related to vocal tract length and skeletal size, affects dominance perceptions [28]. The information obtained from vocal cues can also predict real-world circumstances. In one study, voices of surgeons which were rated as higher in dominance and lower in concern/anxiety, perhaps reflecting an ‘arrogant’ and ‘lack-of-care’ approach, were also more likely to have been previously sued for malpractice, even when controlling for speech content [29]. Likewise, low-pitched CEOs have been shown to manage larger companies and make more money [30].

Vocal parameters, however, are not constant, and can be modulated during social interactions. Shouting during aggressive displays is a typical example, and, in humans and some non-human animals, intensity (loudness) modulations are associated with dominance [27] and hostility [31,32]. Similar to changes in body posture that increase perceived body size, changes in vocal parameters can affect perception of the speaker. Puts et al. [25] reported that men tend to lower their voices during interactions with a competitor when they perceive themselves as physically dominant, and raise it when they believe they are not, exemplifying how elements of self-perceived social status may affect social interactions. Furthermore, taller and more dominant men are less sensitive to visual cues of dominance in other men [33,34], indicating that hierarchical relationships appear to be dependent on perception of relative, rather than absolute, social status, perhaps in an analogous way to how male sensitivity to female attractiveness in humans is stronger towards women of similar, than to lower or higher, mate value [35].

To date, most studies have measured responses to voices with artificially manipulated acoustic parameters (typically F_0_ and D_f_) to investigate how these affect perceptions of dominance [8,25,28,36], but little is known regarding vocal modulations during interactions with dominant or prestigious individuals, particularly in free speech as opposed to individual phonemes or standardised sentences. Although one study examined male responses during interactions depending on their relative physical and social dominance, which in their study was described similarly to our description of prestige [25], whether men and women respond to these two forms of social status in similar ways remains largely unanswered. In our experiment, we aimed to address these questions by measuring within-subject vocal modulations, in both men and women’s voices, in response to dominant, prestigious, or neutral (control) targets. We did this by using a simulated job interview scenario where participants were required to act as a candidate and answer three standardized interview questions (ranging from introductory to interpersonal).

We predicted, first, that participants’ vocal characteristics would change based on whether they were talking to a dominant, prestigious, or neutral target, because signs of social status have been shown to affect vocal characteristics (e.g. [19]), and because dominant individuals appear to be less sensitive to dominance cues in other men [33,34]; and second, that these changes would also be related to the participant’s own self-perceived dominance and prestige. We predicted that those participants rating themselves as more dominant would speak more loudly (i.e. with higher intensity) than those who rated themselves as low in dominance [27], especially when speaking to high-status individuals. Additionally, we expected these high dominance participants to lower their F_0_ when speaking to the dominant target, as these targets may be more likely to be in direct competition with them, for mating opportunities or resources [25]. We had no *a priori* predictions about how participant prestige would affect their interaction with the targets, however as research suggests that both using a dominant or prestigious route leads to attainment of high status, and higher status individuals are more likely to acquire mating opportunities and resources [37,38], there may be reason to expect that responses will be similar with respect to both F_0_ and intensity parameters. On the other hand, if the behaviours of prestigious and dominant individuals differ significantly, then there might be reason to predict that there will be variability like that reported previously (low F_0_ and high intensity) towards dominant individuals but a different pattern of results for prestigious individuals. Additionally, little work has been done on how men and women would differ in their interactions with the male targets. However, recent findings suggest that men and women vary their vocal parameters with respect to the attractiveness of the people they are interacting with [39], perhaps as a strategy to be perceived as more attractive, and as high status men/those with more resources are perceived to be of greater mate value than low status men [40], we predicted that women would vary their F_0_ more towards high status than low status men (but we had no specific predictions for prestige vs. dominance strategies).

Finally, as the three interview questions differed semantically (see full description of questions in methods) we hypothesized that there might be a question effect, with the greatest variation of vocal parameters found in the most interpersonal question (question 3), in which participants would imagine how they might engage with and approach the employer (target) with a problem. That is, those participants rating themselves high in dominance may not vary their F0 to a question simply asking them to introduce themselves, however they may vary (we predict a decrease) their F0 when explaining a situation to/interacting with an employer.

## Materials and methods

### Ethics statement

All procedures obtained ethical approval from the Ethics Committee of the Department of Psychology, Faculty of Natural Sciences, University of Stirling. All participants provided written informed consent and were offered course credit for their participation.

#### Participants

We recruited 48 participants who were students at the University of Stirling (24 men, mean age ± SD = 20.8 ± 6.56; 24 women, 20.2 ± 5.51).

#### Target stimuli

We used EvoFit software [41] to create the face stimuli used in this experiment. This software allows the user to ‘evolve’ a face from sets of available faces over successive iterations, in a holistic (whole face) process as opposed to featurally (adding single features to the face one-by-one). An independent group of 14 men (mean age ± SD = 21.8 ± 7.3) were asked to create same-sex faces using written descriptions of dominant and prestigious individuals based on definitions used in current literature [2,3,42]. Dominant individuals were described as ‘An approximately 36–45 year old male. He is an extremely dominant individual. This person likes to be in control and to get their way. They will use force, coercion, and intimidation to achieve their goals if necessary.’ Prestigious individuals were described as ‘An approximately 36–45 year old male. He is a highly valued, prestigious and influential individual. He has many valued skills and qualities and others follow him freely. This ultimately leads to his achieving his goals.’

These 28 novel faces were rated for dominance and prestige using a 7-point scale (1 = low dominance/prestige; 7 = high dominance/prestige) by 69 undergraduate students (19 men; mean age**±**SD *=* 29.0 **±** 9.7). The two faces which received the highest dominance (mean **±** SD *=* 5.1 **±** 1.3) and highest prestige (mean **±** SD = 3.99 **±** 1.3) scores were used as stimuli (i.e., as the dominant and prestigious employers, respectively). For the ‘neutral’ employer, the face receiving the median rating on dominance (mean **±** SD = 3.3 **±** 1.3) and prestige (mean **±** SD = 3.1 **±** 1.3) was used.

**Fig 1:**
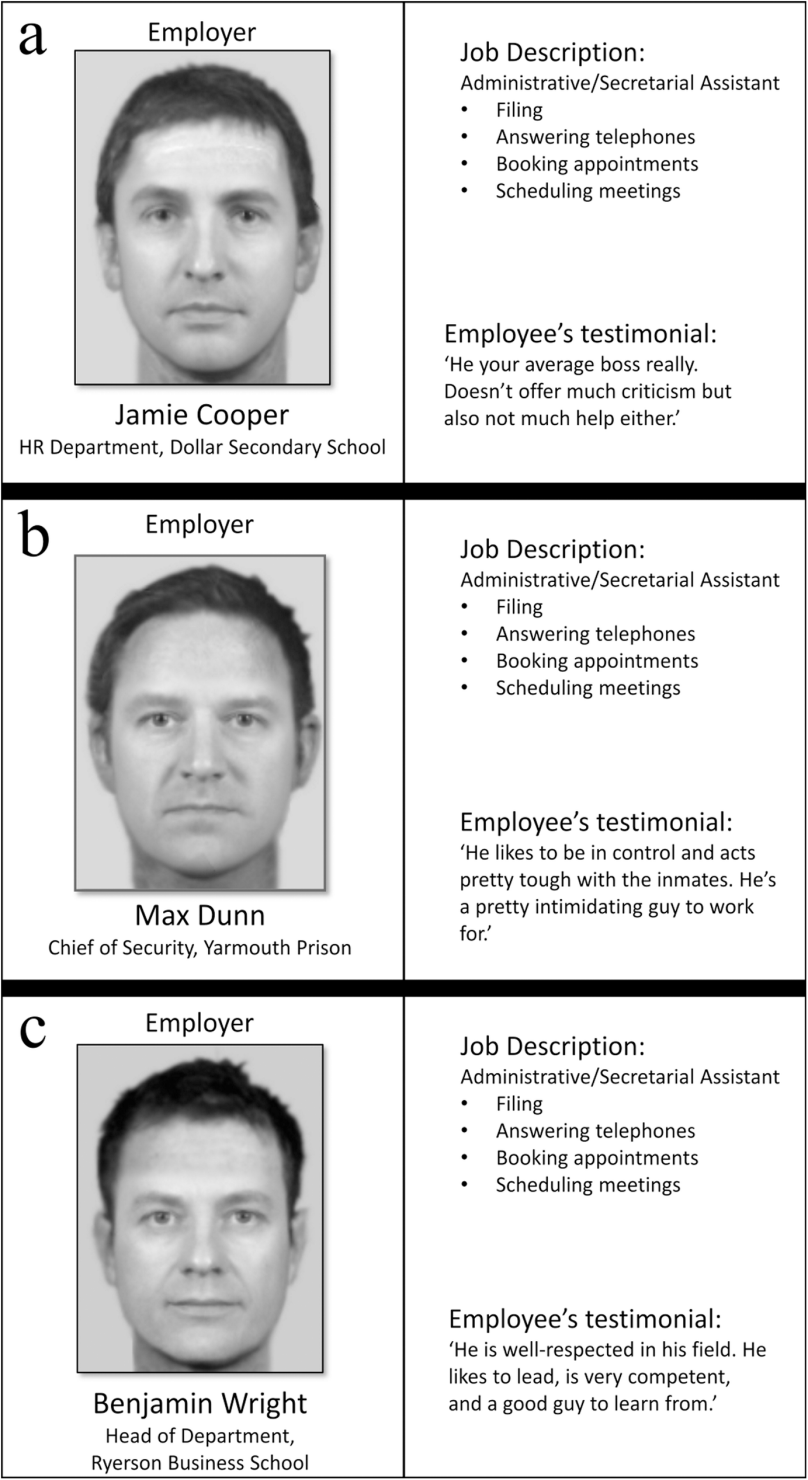
Final targets as presented to participants. **a)** neutral target; **b)** dominant target; **c)** prestigious target. All targets include facial images, names, job titles and employee testimonials.

We then created three different ‘employer profiles’, which contained a face image and text description, including a name, a job title, and an employee testimonial. The name, job title and testimonial were used to further manipulate the impression of targets as either dominant, prestigious, or neutral (Fig 1 shows the three profiles). The three profiles were also scored by an independent group of raters (see Target Stimuli in S1 Text) for prestige and dominance, confirming that in all cases the attributes of the dominant target were rated as more dominant, the attributes of the prestigious target as more prestigious, and the attributes for the neutral target were rated as neither high in dominance or prestige; faces were additionally rated for perceived attractiveness and age (results of these ratings are presented in S1 Table and S1 Fig). Finally, job descriptions were identical (i.e. administrative/secretarial assistant including filing, answering telephones, booking appointments and scheduling meetings).

#### Experimental procedure

Participants were first told that the ‘experiment’ they were participating in was in fact a ‘pilot’ to test the effectiveness of a new interviewing technique which did not require the interviewee and interviewer to be in the same room. After written informed consent was obtained, participants were presented with the experiment using Qualtrics software (Qualtrics, Provo, UT, 2013; www.qualtrics.com), on a desktop computer located in a quiet room. Monaural audio responses of the participants were digitally recorded using Praat 5.2.44 (P. Boersma and D. Weenink, 2011; www.praat.org), with a sampling frequency of 44.1 kHz, using a head mounted microphone positioned about 2 cm from the participant’s mouth. Additionally, participants were video recorded to emphasise monitoring and assessment of behaviour as in real-world interviews; the experimenters highlighted the recordings by adjusting the videorecorder in the presence of participants, while they viewed a real-time recording of themselves on a monitor.

To control for any potential order effects, 24 male and 24 female participants were shown the three targets in one of six possible sequences (i.e. 1: Dominant (D)-Prestigious (N)-Neutral (N); 2: D-N-P; 3: P-D-N; 4: P-N-D; 5: N-D-P; 6: N-P-D; the sequences were counterbalanced across participants). For each of the three targets, participants were asked to record responses to three common interview questions; hence we recorded 9 instances of speech from each participant. The interview questions were: 1) ‘please introduce yourself to this potential employer in a few sentences’, 2) ‘please tell this employer why you are a good candidate for the job’, and 3) ‘if you had a problem with a colleague at work how would you convey it to your boss?’. Aside from the generic nature of the questions, they were also selected to differ in their interpersonal characteristics. That is, while question 1 was purely a request for the subject to introduce themselves, question 2 added a personal component in requiring the participant to think about and articulate what personal attributes they believed would make them qualified for the job. Finally, question 3 had an interpersonal emphasis and required the participant to think about how they might engage with and approach the employer (target) with a problem. Although a simulated job interview may not completely reflect a real-life situation, mock interviews have been shown to increase anxiety levels before and during the interview [43] and in our design participants were aware of being video and audio recorded to increase the realism of the scenario.

After recording their responses, participants were asked to enter some basic demographic information, fill in a self-report scale of dominance and prestige [3], rate the dominance and prestige of the three targets, and explain what they thought the purpose of the study was (see Experimental Procedure in S1 Text). The entire experiment was presented using Qualtrics software, and was completed by participants while they were alone in a room. Once they had finished the experiment, participants were debriefed, given the opportunity to ask any remaining questions, and were asked to confirm whether they still consented to the use of their data.

In total, 429 recordings were obtained (3 were discarded due to background noise that affected audio quality), with length ranging from 4 to 107 seconds (mean **±** SD = 25.02 **±** 16.41s). Length of recording did not differ significantly depending on which target participants were responding to (repeated-measures GLM: *F*_2, 86_ = 0.95, *p* = .39).

#### Manipulation check

**Fig 2:**
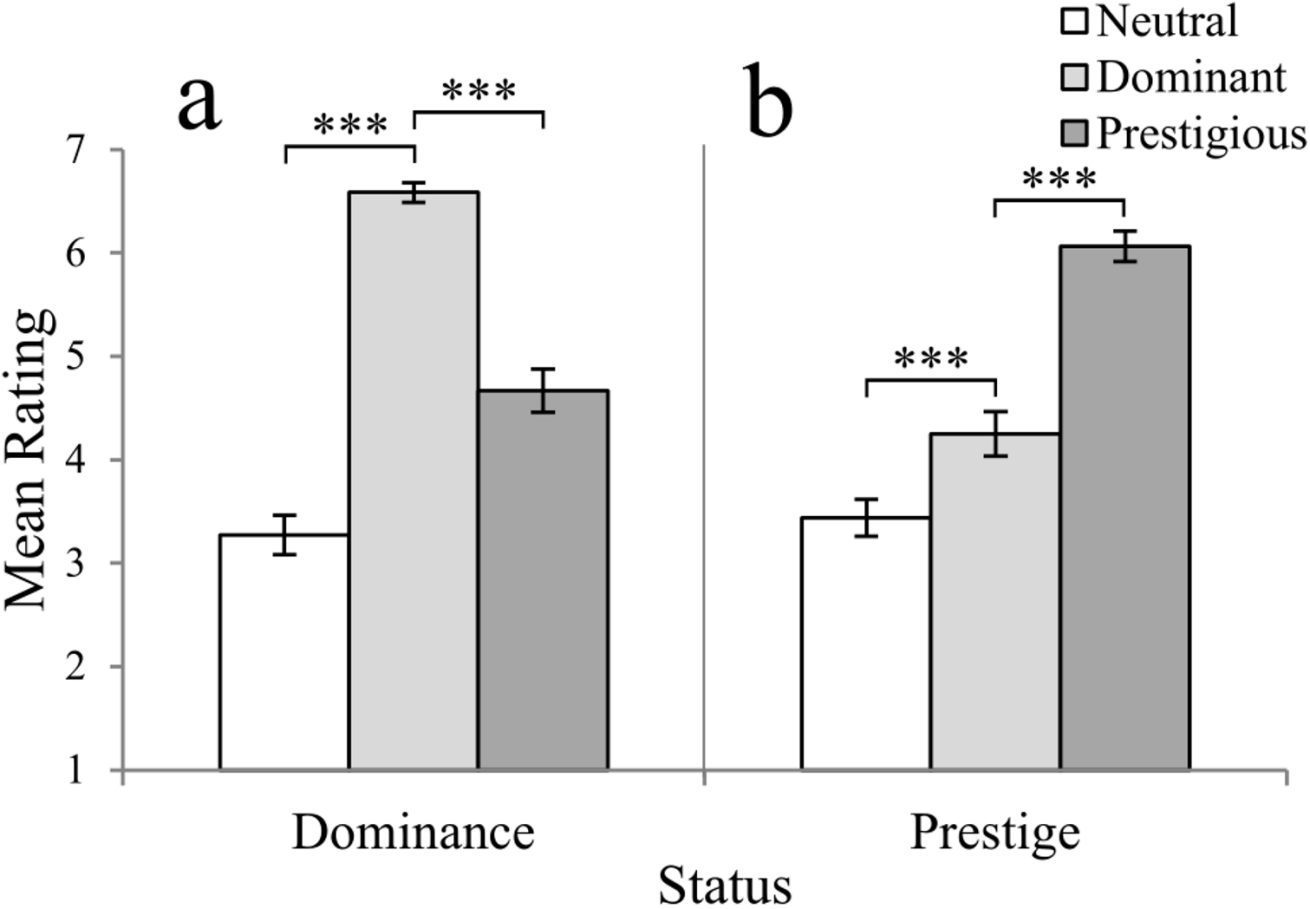
Ratings of the final target profiles given by participants. **a)** dominance rating; **b)** prestige rating. Neutral: white bars; dominant: light grey bars; prestigious: dark grey bars. For pairwise comparisons, *** *p* < .001 (d.f. = 47 in all cases). Bars represent estimated marginal means **±** 1 s.e.m.

As a final step, we conducted a manipulation check. Once participants had completed the experiment, we asked them to rate the full profiles for prestige and dominance. These ratings confirmed that the mean dominance rating of the dominant target (mean ± *SD =* 6.58 ± 0.65) was significantly higher than the ratings of both the prestigious (mean ± *SD* = 4.66 ± 1.46) and neutral (mean ± *SD* = 3.27 ± 1.32) targets (*F*_2,94_ = 87.99, *p* < .001; Fig 2A), and the prestigious target was rated as more prestigious (mean ± *SD* = 6.06 ± 1.04) than the dominant (mean ± *SD* = 4.25 ± 1.49) and neutral (mean ± *SD* = 3.44 ± 1.22) targets (*F*_2,94_ = 57.62, *p* < .001; Fig 2B).

#### Data analysis

We analysed each recording using Praat, obtaining values every 10 ms on intensity (dB) and F_0_ (Hz). F_0_ was measured using a noise-resistant autocorrelation method, between 75 and 300 Hz for male voices, and 100 and 500 Hz for female voices, as recommended by the software programmers. To ensure that intensity values were not affected by differences in the length or number of silent periods, and to control for background noise during these, we only used values which corresponded to times points in which the Praat algorithm produced a value of pitch.

For the statistical analysis, we calculated five variables from each of the 429 recording (9 recordings per participant), two of which were related to intensity: mean intensity and intensity variability (intensity SD), and three to F_0_: mean F_0_, F_0_ variability (F_0_ SD), and minimum F_0_. These final values were analysed using repeated-measures general linear models (GLM) for each parameter (with Holm-Bonferroni [44] adjustments for multiple tests, because we performed two analyses of intensity parameters, and three of F_0_ parameters), using sex of the participant (PS) as a between-subject factor, target and question as within-subject factors, and participant dominance (PD) and participant prestige (PP) as covariates.

Because both PD and PP are time-invariant, thus in order to use them as covariates, we compared the effect of the target on the vocal parameters of the participants, centring the covariate values to their mean [45–47]. First, we created a model including both PD and PP as covariates, for each dependent variable (each acoustic parameter). Significant interactions between a covariate and a within-subject factor, suggest that there is a difference in the slope of the regression relating the covariate to the dependent variable, for each level of the within-subject factor; for example, a significant interaction between target (neutral, dominant, prestigious) and PD, represents that there are different regression slopes of PD and the studied acoustic parameter, for each target. Then, for covariates significantly interacting with within-subject factors, we performed further analyses including only one covariate (PD or PP), centred to the mean, as well as low and high values (10^th^, and 90^th^ percentiles). In such cases, comparing the main effect of a within-subject factors across different levels of the covariate is of particular interest [46], as it shows predicted values for the dependent variable, for participants on different levels of the covariate (low, mean and high self-rated prestige or dominance). Self-rated PD ranged from 1.5 to 5.0, and the mean, 10^th^, and 90^th^ percentiles were 2.89, 1.74, and 4.00 respectively; equivalent values for PP, which ranged from 3.56 to 6.44, were 4.73, 3.78, and 5.69. All tests are two-tailed.

While statistical analyses were performed using acoustical data (in dB and Hz for intensity and F_0_ parameters, respectively), most figures were created using standardised values (z scores) for each participant to account for between-subject differences (most noticeably sex differences in F_0_ as well as F_0_ SD [39]) and represent within-subject trends.

## Results

First, we tested whether individuals’ self-rated status (prestige and dominance) predicted their vocal parameters, in response to each target. Then we tested if individuals altered their vocal parameters in speech directed at dominant or prestigious individuals. We conducted separate analyses testing within-subject differences in parameters related to intensity (mean intensity and intensity SD) and F_0_ (mean F_0_, F_0_ SD, and minimum F_0_), with planned contrasts (Helmert) comparing responses to the neutral versus the high-status targets (dominant and prestigious), and between the two high-status targets (dominant versus prestigious). Descriptive statistics of the acoustic vocal parameters of the responses of the participants to each type of target are presented in S2 Table.

### Relationships between vocal parameters and self-rated status

As we predicted participants would adjust their vocal characteristics based on their self-rated status (prestige and dominance), in our analyses we used these self-ratings as covariates, and tested whether there were relationships between each acoustic parameter, in response to each target, and the participants’ own ratings of dominance (PD) and prestige (PP; Table 1). Mean (**±** SD) self-rated scores of PD were 3.07 **±** 0.56 and 2.71 **±** 0.91 for men and women, respectively; scores for PP were 4.66 **±** 0.59 and 4.79 **±** 0.83. As there were no significant differences in PD or PP between men and women (*t*-tests: PD: *t*_46_ = 0.63, *p* = .11; PP: *t*_46_ = 1.67, *p* = .53), we pooled these data in the analyses below.

**Table 1:** Correlations between vocal parameters in responses to each target and participants’ status.

	Mean intensity			Intensity SD			Mean F0			F0 SD			Min F0		
	N	D	P	N	D	P	N	D	P	N	D	P	N	D	P
PD	.073	-.012	.056	.059	.012	-.005	-.276*	-.335**	-.347**	**-.193†**	-.295*	-.253*	**-.207†**	-.260*	**-.239†**
PP	-.005	-.006	.006	**-.239†**	-.254*	**-.232†**	-.073	-.071	-.081	.043	.065	.106	.045	.058	-.029

PD = Participant Dominance, PP = Participant Prestige. Results are from correlations for the responses to each target (N = neutral, D = dominant, P = prestigious) with participants' status (PD, PP), for each vocal parameter. Results reported in this table are Spearman’s *ρ* (*n* = 48 in every case).

†*p* < .10

*p < .05

**p < .01.

As expected, participants who rated themselves as higher in dominance had lower F_0_, as well as lower F_0_ SD, and minimum F_0_, although these trends did not reach significance in all cases. There was also a trend for more prestigious participants to vary their intensity less, particularly when responding to the dominant target.

### Intensity parameters

Previous research showed that voices with higher mean amplitude and amplitude SD (amplitude is directly proportional to intensity) are perceived as more dominant [27]. Because of this, we anticipated that participants would adjust the intensity of their voices depending on the perceived status (dominance or prestige) of the targets, and their self-perceived dominance (PD) and prestige (PP). However, the analysis of intensity parameters revealed no significant differences in the mean intensity or intensity SD of the participants’ responses depending on the target, even when including PP and PD (as covariates, centred to their mean), nor a significant interaction between participant sex and target (for detailed results, see S3 Table).

### Fundamental frequency (F_0_) parameters

The analysis of F_0_ parameters revealed that mean F_0_ was particularly sensitive to our manipulation (Table 2). Although the main effect of target did not reach significance, it showed a trend in which the mean F_0_ of the participants progressively increased in responses to the neutral (mean ± s.e.m. = 156.17 ± 2.05 Hz), dominant (156.75 ± 2.27 Hz), and prestigious (157.10 ± 2.25 Hz) targets (Table 2). When including PD and PP as covariates, the interaction between target and PD did reach significance (*p* = .01), suggesting that the slope of the regression relating PD to F_0_, differs between the different targets (Table 2).

**Table 2:** Context-dependent variation in vocal parameters related to F_0_.

Within-subject Effect	Mean F_0_			F_0_ SD			Min F_0_		
	F	d.f.	p	F	d.f.	p	F	d.f.	p
T	0.28	2, 86	.759	0.08	1.64, 70.64*	.894	1.45	2, 86	.240
T * PD	**4.94**	**2, 82**	**.013**	1.14	1.65, 67.48*	.325	2.34	2, 82	.103
T * PP	0.21	2, 82	.813	0.63	1.65, 67.48*	.535	0.43	2, 82	.654
T * PS	0.57	2, 86	.567	2.42	1.64, 70.64*	.106	0.62	2, 86	.543
Q	**21.21**	1.45, 62.44*	**< .001**	4.55	2, 86	.013	0.59	2, 86	.558
Q * PD	0.40	1.50, 61.60*	.670	1.39	2, 82	.255	2.64	2, 82	.078
Q * PP	**6.43**	1.50, 61.60*	**.003**	2.61	2, 82	.080	0.02	2, 82	.977
Q * PS	**11.67**	1.45, 62.44*	**< .001**	**14.49**	**2, 86**	**< .001**	1.51	2, 86	.226
T * Q	1.64	2.58, 110.87*	.191	0.86	4, 172	.487	1.06	2.49, 107.27*	.363
T * Q * PD	**3.99**	2.72, 111.33*	**.004**	1.27	4, 164	.284	2.41	2.52, 103.13*	.051
T * Q * PP	1.41	2.72, 111.33*	.232	0.53	4, 164	.711	0.72	2.52, 103.13*	.578
T * Q * PS	1.17	2.58, 110.87*	.320	1.01	4, 172	.404	1.19	2.49, 107.27*	.314

T = Target (neutral, dominant, prestigious), Q = Question, PD = Participant Dominance, PP = Participant Prestige, PS = Participant Sex (male, female). Results are from repeated-measures general linear models for each vocal parameter, with Holm–Bonferroni adjustment for multiple tests. Significant effects are in bold.

*Sphericity could not be assumed and Greenhouse–Geisser correction was used.

Interactions with a covariate (PD, PP) are taken from the ANCOVA. All other effects are taken from an ANOVA (see [41]) on the same data without the covariates. For all results, including between-subject effects, see S3 Table.

**Fig 3:**
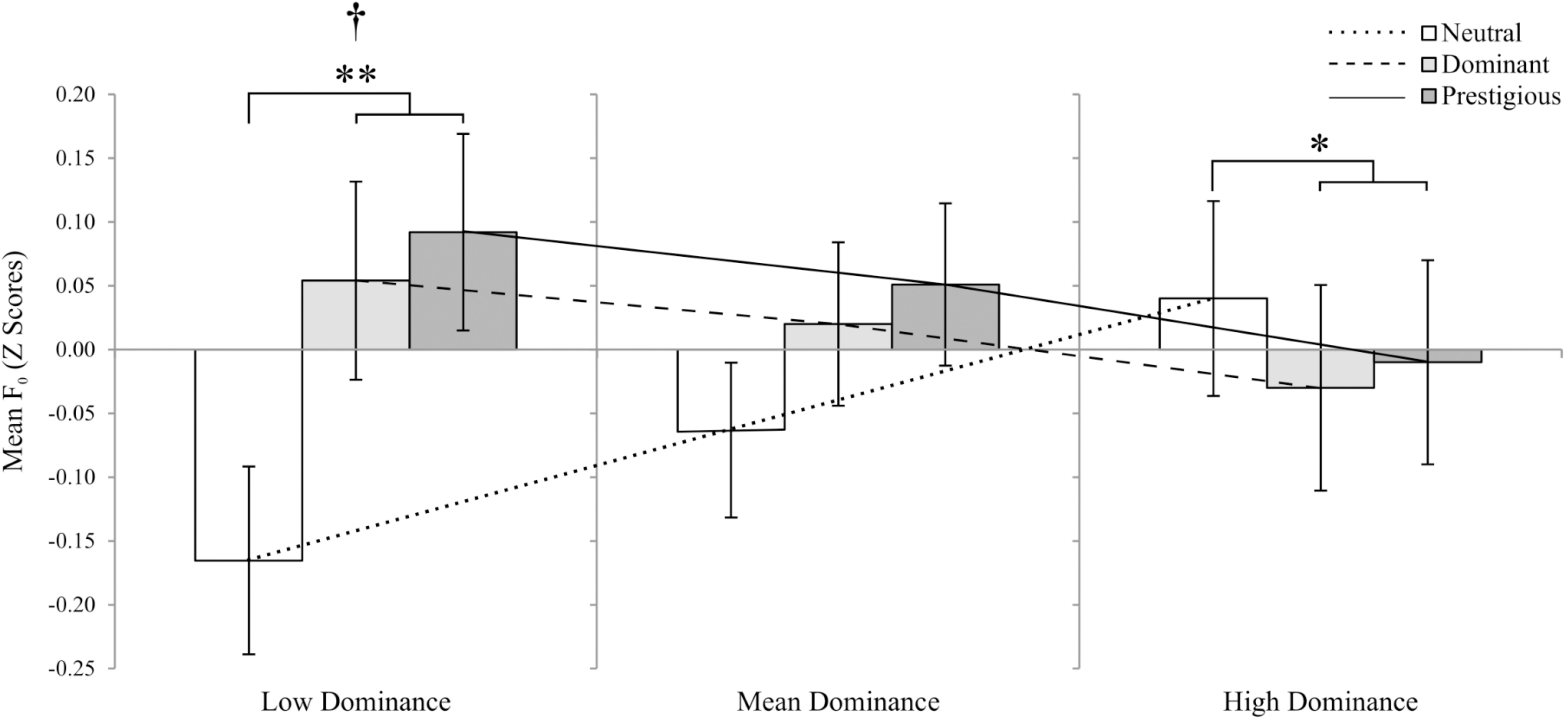
Mean F_0_ in responses the three targets. Mean F_0_ estimated for participants with varying degrees of dominance (Low dominance = 10^th^ percentile; High dominance = 90% percentile. Neutral target: white bars; dominant target: light grey bars; prestigious target: dark grey bars. † represents a main effect of target. For panned contrasts (Table 3), shapes above the bars represent a significant difference between responses to neutral versus high-status targets (dominant and prestigious). *p < .05, **p < .01. Bars represent estimated marginal means ± 1 s.e.m. Straight lines represent linear regressions for responses to each type of target, from which marginal means are estimated (neutral: dotted line; dominant: dashed line; prestigious: solid line). In all cases, F_0_ values were standardised (to z scores) for each participant to make results equivalent and account for between-subject differences.

Centring PD to the mean, 10^th^ and 90^th^ percentile, revealed differences in F_0_ according to who the target participants were responding to, for participants with low self-perceived dominance (*F*_2,82_ = 4.56, *p =* .01), but not for participants with mean (*F*_2,82_ = 0.81, *p* = .45), or high (*F*_2,82_ = 0.75, *p* = .48) self-perceived dominance. This suggests that only individuals who perceive themselves as low in dominance modulate their voices, depending on the status of the person they are speaking to (Fig 3).

**Fig 4:**
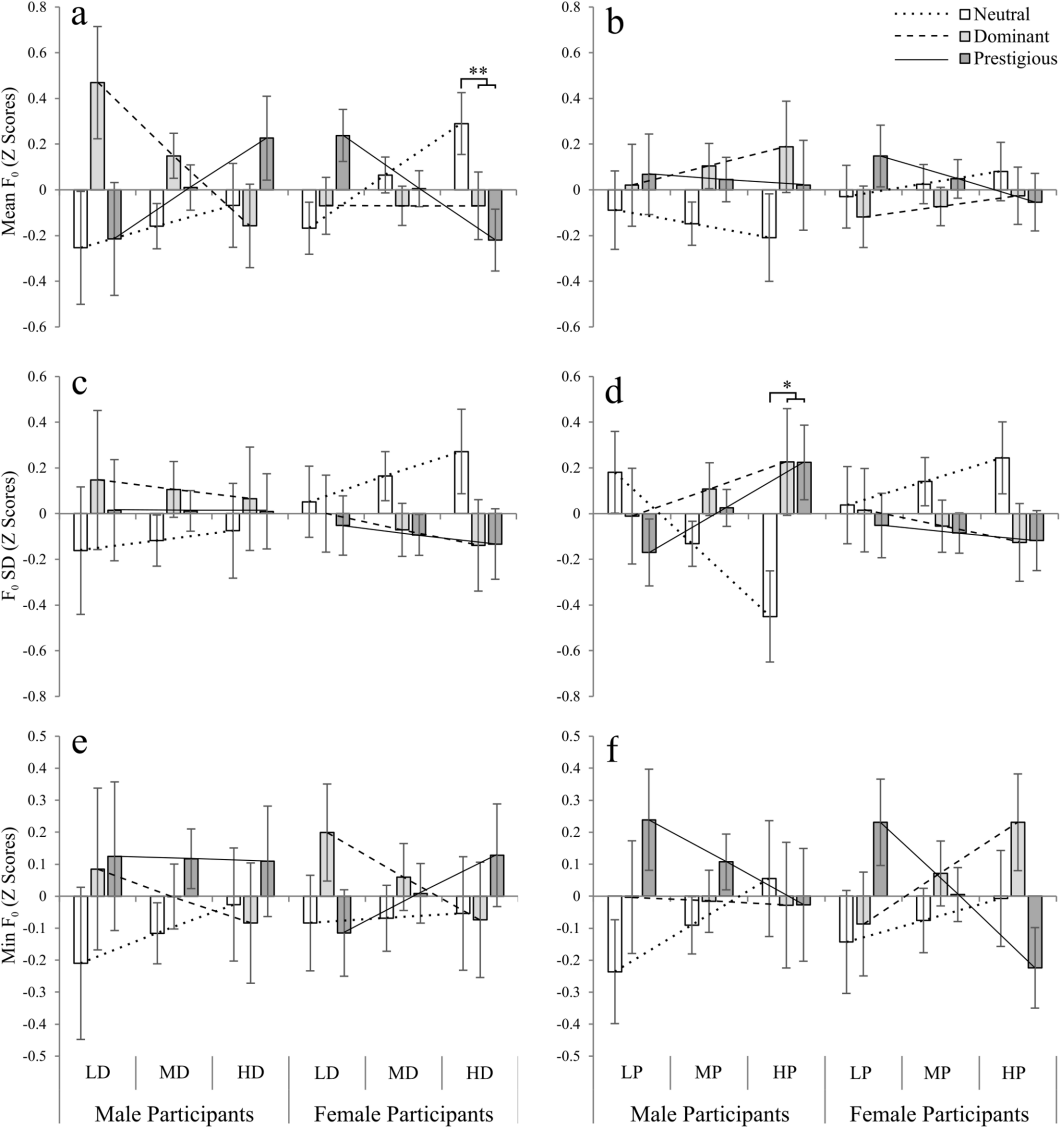
Modulation of vocal parameters related to F_0_ in speech towards the three targets estimated for participants with varying degrees of dominance. **a, b)** Mean F_0_; **c, d)** F_0_ SD; **e, f)** Minimum F_0_. Neutral target: white bars; dominant target: light grey bars; prestigious target: dark grey bars. Results are split by sex of the participants, and estimated for participants with low (10th percentile), mean, and high (90% percentile) dominance (panels a, c, e) and prestige (panels b, d, f). LD = low dominance; MD = mean dominance; HD = high dominance; LP = low prestige; MP = mean prestige; HP = high prestige. Standard deviation (SD) was used as a measure of variability. Results were standardised (to z scores) for each participant to make results equivalent and account for between-subject’s differences. For panned contrasts (Table 3), shapes above the bars represent a significant difference between responses to neutral versus high-status targets (dominant and prestigious). *p < .05, **p < .01. Bars represent estimated marginal means ± 1 s.e.m. Straight lines represent linear regressions for responses to each type of target, from which marginal means are estimated (neutral: dotted line; dominant: dashed line; prestigious: solid line).

Planned contrasts, however, revealed that in the case of the main effect of target, there was a significant difference in the mean F_0_ of the participants between the neutral versus the high-status targets (dominant, prestigious), but not between the two high-status targets (dominant versus prestigious; Table 3), predicted for participants with low self-perceived dominance as well as high self-perceived dominance (Fig 3); participants who perceived themselves as low in dominance tended to speak with a higher mean F_0_ towards high-status targets, in comparison to the neutral target, while the opposite trend was found for high self-perceived participants. No such tendency was found for participants with mean dominance. Similarly, for the interaction between target and participant sex, F_0_ SD was significantly different when including PD as a covariate, and comparing responses to the neutral and high-status targets (*F*_1, 41_ = 5.65, *p* = .02), but not between the two high-status targets (Fig 4C, S4 Table). Thus, it appears that women varied F_0_ more when talking to neutral targets than dominant and prestigious targets, while the opposite effect was evident in men: they varied their F_0_ less when speaking to neutral targets than dominant and prestigious targets.

**Table 3:** Planned contrasts estimated for participants with varying degrees of dominance and prestige.

Effect	Planned Contrasts	Mean F_0_						F_0_ SD						Min F_0_					
		F	p	F	p	F	p	F	p	F	p	F	p	F	p	F	p	F	p
PD		Low		Mean		High		Low		Mean		High		Low		Mean		High	
T	N vs HS	**9.13**	**.004**	0.80	.375	**4.61**	**.038**	1.37	.249	0.01	.935	1.72	.197	0.01	.913	0.79	.378	1.24	.272
	D vs P	0.78	.381	0.15	.703	0.24	.625	0.32	.574	0.15	.700	0.02	.877	0.65	.424	1.59	.214	**5.10**	**.029**
T * Q	N vs HS	0.33	.570	2.86	.098	**6.23**	**.017**	1.24	.272	1.07	.307	**5.38**	**.025**	2.88	.097	1.70	.200	0.10	.758
	D vs P	1.85	.181	0.19	.662	3.65	.063	1.30	.260	0.06	.809	0.84	.364	0.69	.410	1.01	.321	0.07	.797
PP		Low		Mean		High		Low		Mean		High		Low		Mean		High	
T	N vs HS	0.92	.342	0.43	.516	0.03	.856	0.05	.829	0.03	.872	0.17	.679	0.11	.745	0.86	.359	0.62	.434
	D vs P	0.49	.490	0.12	.736	0.09	.769	1.42	.241	0.14	.708	0.56	.458	1.56	.219	1.77	.191	0.12	.733
T * Q	N vs HS	0.15	.700	3.20	.081	**6.53**	**.014**	0.10	.759	1.21	.278	1.03	.315	3.96	.053	1.58	.216	0.25	.622
	D vs P	0.14	.712	0.28	.599	1.03	.315	0.81	.373	0.03	.860	0.49	.489	1.06	.310	0.98	.327	0.02	.875

T = Target (neutral, dominant, prestigious), Q = Question, N = Neutral Target, HS = High-status Targets (dominant, prestigious), D = Dominant Target, P = Prestigious Target. For participants, self-perceived status covariates (PD = Participant Dominance, PP = Participant Prestige) were centred to low (10th percentile), mean, and high (90% percentile) levels. Results are from planned contrasts (Helmert) for each vocal parameter (d.f. = 1, 41), including only PD or PP as a covariate. All values are taken from an ANCOVA. Significant effects are in bold. Of particular interest, is the main effect of target (T), and its changes for participants with different levels of dominance or prestige. For all results, including between-subject effects and interactions, see S4 Table.

In addition, the general analysis and planned contrasts revealed the importance of the effects of question in the vocal parameters of spoken responses: there was a significant main effect of question, as well as significant interactions between question and PP on the mean F_0_ of the participants (Table 2), and a significant interaction between question and participant sex for both mean F_0_ and F_0_ SD (Table 2); furthermore, the interaction between target, question and PD was significant, suggesting that the specific characteristics of the questions (introductory, personal, interpersonal) had an effect on the vocal parameters of the responses (Table 2). Planned contrasts revealed that in the cases of the interactions between target and question (for mean F_0_, including either PD or PP as covariate), there was a significant difference between the neutral versus and high-status targets, but not between the high-status targets, for participants with high PD and high PP (Table 3).

### Analysis of Fundamental frequency (F_0_) parameters by question

Paralinguistic parameters thus vary depending on the target and self-rated status of the speaker, but participants changed their vocal characteristics of their responses according to the question they were responding to. To further explore this connection, we split the analysis by question in order to test the effect that the specific context of each question had on the responses.

**Fig 5:**
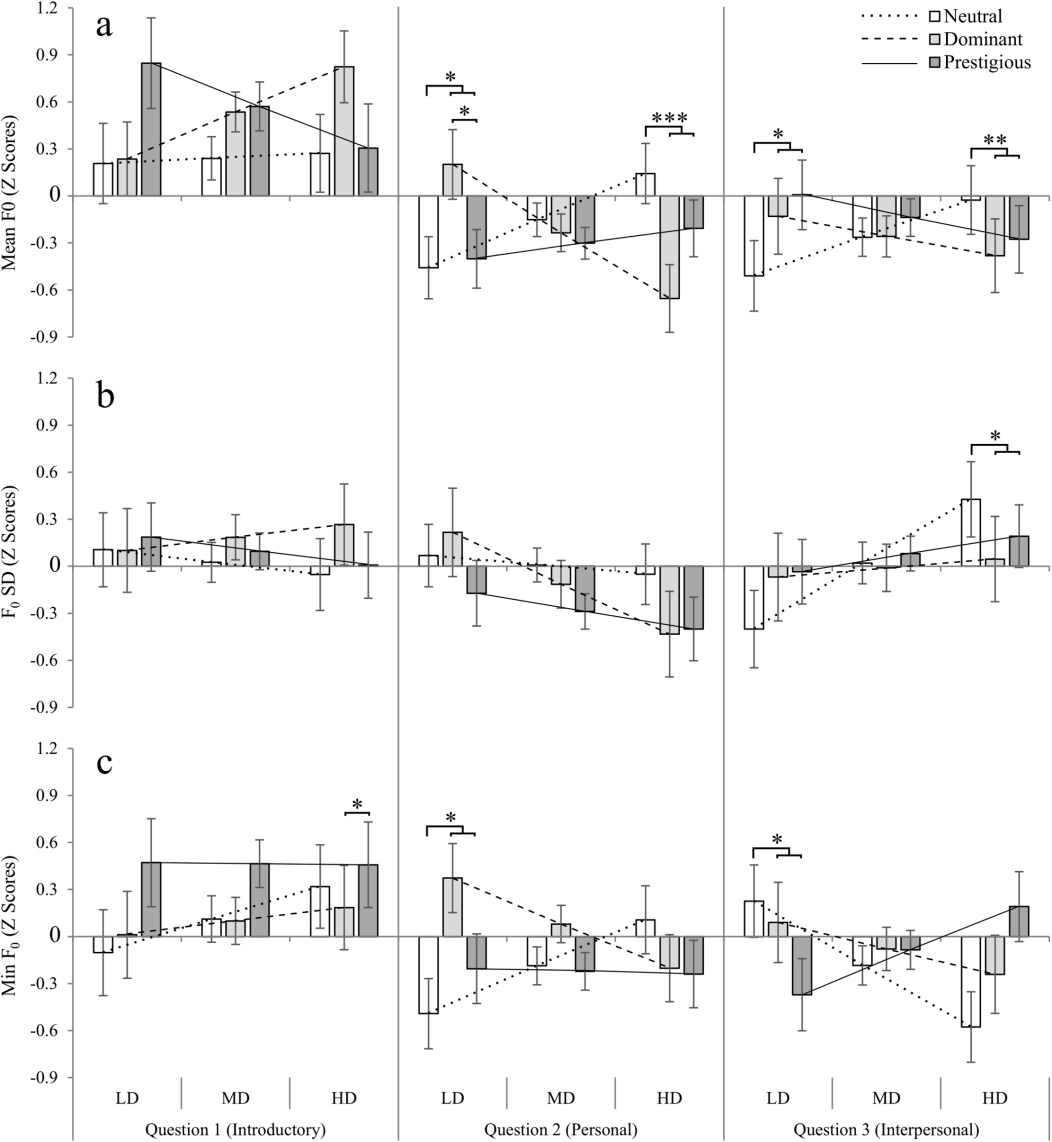
Modulation of vocal parameters related to F_0_ in speech towards the three targets estimated for participants with varying degrees of dominance, by question. **a)** Mean F_0_; **b)** F_0_ SD; **c)** Minimum F_0_. Neutral target: white bars; dominant target: light grey bars; prestigious target: dark grey bars. Results are split by question (left: Question 1 –Introductory; centre: Question 2 –Personal; right: Question 3 –Interpersonal), and estimated for participants with low (10th percentile), mean, and high (90% percentile) dominance. LD = low dominance; MD = mean dominance; HD = high dominance. Standard deviation (SD) was used as a measure of variability. Results were standardised (to z scores) for each participant to make results equivalent and account for between-subject’s differences. For planned contrasts (Table 5), double shapes above the bars represent a significant difference between responses to neutral versus high-status targets (dominant and prestigious), and single shapes represent a significant difference between responses to dominant versus prestigious targets. *p < .05, **p < .01, ***p < .001. Bars represent estimated marginal means ± 1 s.e.m. Straight lines represent linear regressions for responses to each type of target, from which marginal means are estimated (neutral: dotted line; dominant: dashed line; prestigious: solid line).

This analysis revealed that in the case of question 1 (Introductory), there were no significant differences in the vocal parameters of the participants depending on the target they were responding to (Table 4 and Fig 5).

**Table 4:** Context-dependent variation in vocal parameters related to F_0_ by question.

Effect	Mean F_0_			F_0_ SD			Min F_0_		
	F	d.f.	p	F	d.f.	p	F	d.f.	p
Question 1 (Introductory)									
T	1.21	2, 88	.303	0.50	2, 88	.610	2.59	1.58, 69.32*	.094
T x PD	2.36	2, 84	.101	0.29	2, 84	.748	1.63	1.60, 67.24*	.207
T x PP	0.08	2, 84	.451	0.12	2, 84	.890	0.71	1.60, 67.24*	.464
T x PS	0.24	2, 88	.784	0.18	2, 88	.833	0.39	1.58, 69.32*	.631
Question 2 (Personal)									
T	0.56	1.70, 76.42*	.546	1.10	1.52, 68.41*	.326	0.96	2, 90	.389
T x PD	**8.55**	1.75, 75.18*	**< .001**	0.43	1.52, 65.46*	.597	1.44	2, 84	.243
T x PP	1.59	1.75, 75.18*	.213	0.15	1.52, 65.46*	.802	0.34	2, 84	.716
T x PS	0.41	1.70, 76.42*	.633	1.03	1.52, 68.41*	.346	**0.31**	**2, 90**	**.049**
Question 3 (Interpersonal)									
T	1.58	2, 92	.212	0.15	1.74, 79.90*	.833	0.44	2, 92	.648
T x PD	**6.06**	**2, 84**	**.003**	2.94	1.74, 76.47*	.066	**3.69**	**2, 84**	**.029**
T x PP	0.90	2, 84	.411	1.31	1.74, 76.47*	.274	0.92	2, 84	.404
T x PS	**3.64**	**2, 92**	**.034**	3.31	1.74, 79.90*	.075	0.16	2, 92	.857

T = Target (neutral, dominant, prestigious), PD = Participant Dominance, PP = Participant Prestige, PS = Participant Sex (male, female). Results are from repeated measures general linear models for each vocal parameter. Significant effects are in bold.

*Sphericity could not be assumed and Greenhouse–Geisser correction was used.

Interactions with a covariate (PD, PP) are taken from the ANCOVA. All other effects are taken from an ANOVA [see, 41] on the same data without the covariate.

However, participants responding to questions 2 (Personal) and 3 (Interpersonal), showed a significant interaction between target and participant dominance for F_0_. In short, this means that the slope of the association between PD and F_0_, was different for the three targets (Table 4 and Fig 5A). Planned contrasts revealed that in responses to questions 2 (Personal) and 3 (Interpersonal), mean F_0_ was significantly higher when responding to high-status versus neutral targets for participants with low self-perceived dominance, while no such difference existed for participants with mean PD, and participants who perceive themselves as high in dominance showed the opposite trend: they had a lower F_0_ in responses to high-status targets, than to the neutral target (Table 5 and Fig 5A). Similarly, participants with high PD, tended to speak with higher F_0_ SD to the neutral than the high-status targets in question 3, while the opposite trend was found (though not significant), for participants with low PD (Table 5 and Fig 5B). In terms of minimum F_0_, in question 3 participants with low PD reached a significantly lower F_0_ when responding to high-status than the neutral target (Table 5 and Fig 5C).

**Table 5:** Planned contrasts for variation in vocal parameters related to F_0_.

**Effect**	**Planned Contrasts**	**Mean F**_**0**_						**F**_**0**_ **SD**						**Min F**_**0**_					
		***F***	***p***	***F***	***p***	***F***	***p***	***F***	***p***	***F***	***p***	***F***	***p***	***F***	***p***	***F***	***p***	***F***	***p***
PD		Low		Mean		High		Low		Mean		High		Low		Mean		High	
Question 1 (Introductory)																			
T	N vs HS	0.84	.365	3.02	.090	0.98	.328	0.26	.612	1.05	.310	0.38	.541	0.03	.862	1.34	.254	1.21	.277
	D vs P	3.22	.080	0.01	.932	3.01	.090	0.37	.547	0.00	.952	0.31	.583	0.06	.809	3.19	.081	**4.96**	**.031**
Question 2 (Personal)																			
T	N vs HS	**6.84**	**.012**	0.95	.336	**13.80**	**.001**	0.03	.856	2.11	.153	2.02	.163	**4.58**	**.038**	1.82	.184	0.45	.506
	D vs P	**4.09**	**.049**	0.30	.590	2.08	.156	0.47	.498	0.06	.809	0.18	.675	0.11	.738	0.09	.769	0.00	.989
Question 3 (Interpersonal)																			
T	N vs HS	**6.19**	**.017**	0.33	.568	**9.58**	**.003**	2.98	.091	0.21	.651	**4.88**	**.032**	**5.70**	**.021**	0.87	.355	1.81	.185
	D vs P	0.45	.507	3.85	.056	2.19	.146	0.24	.626	0.10	.759	0.68	.414	1.70	.199	0.14	.715	2.87	.097
**Effect**	**Planned Contrasts**	**Mean F**_**0**_						**F**_**0**_ **SD**						**Min F**_**0**_					
		***F***	***p***	***F***	***p***	***F***	***p***	***F***	***p***	***F***	***p***	***F***	***p***	***F***	***p***	***F***	***p***	***F***	***p***
PP		Low		Mean		High		Low		Mean		High		Low		Mean		High	
Question 1 (Introductory)																			
T	N vs HS	0.01	.935	3.14	.083	**4.14**	**.048**	0.16	.692	1.06	.308	0.69	.410	1.53	.223	1.38	.247	0.03	.871
	D vs P	0.45	.506	0.00	.992	0.44	.513	0.05	.831	0.00	.970	0.07	.796	2.66	.110	3.25	.079	0.27	.606
Question 2 (Personal)																			
T	N vs HS	0.83	.366	0.88	.352	**4.22**	**.046**	0.14	.711	2.14	.151	1.89	.176	1.77	.190	1.65	.206	0.04	.850
	D vs P	0.01	.931	0.22	.642	0.42	.519	0.02	.903	0.05	.822	0.02	.884	0.46	.501	0.09	.767	0.11	.741
Question 3 (Interpersonal)																			
T	N vs HS	0.72	.401	0.29	.594	2.22	.143	0.03	.872	0.19	.664	0.13	.723	3.37	.073	0.82	.370	0.60	.444
	D vs P	1.13	.292	3.84	.056	1.58	.215	1.42	.240	0.10	.751	2.49	.122	0.03	.873	0.13	.720	0.07	.791

T = Target (neutral, dominant, prestigious), N = Neutral Target, HS = High-status Targets (dominant, prestigious), D = Dominant Target, P = Prestigious Target. For participants, self-perceived status covariates (PD = Participant Dominance, PP = Participant Prestige) were centred to low (10th percentile), mean, and high (90% percentile) levels. Results are from planned contrasts (Helmert) for each vocal parameter (d.f. = 1, 43; 1, 44; and 1, 45 for question 1, 2, and 3 respectively), including only PD or PP as a covariate. All values are taken from an ANCOVA. Significant effects are in bold. Of particular interest, are changes is the main effect of target (T), for participants with different levels of dominance or prestige. For all results, including between-subject effects and interactions, see S5 Table.

Additionally, responses to question 3 (Interpersonal) were significantly different for male and female participants depending on the target: while the mean F_0_ of male participants was lower in responses to the neutral target, female participants had lower mean F_0_ in responses to the dominant target (Table 4).

**Fig 6:**
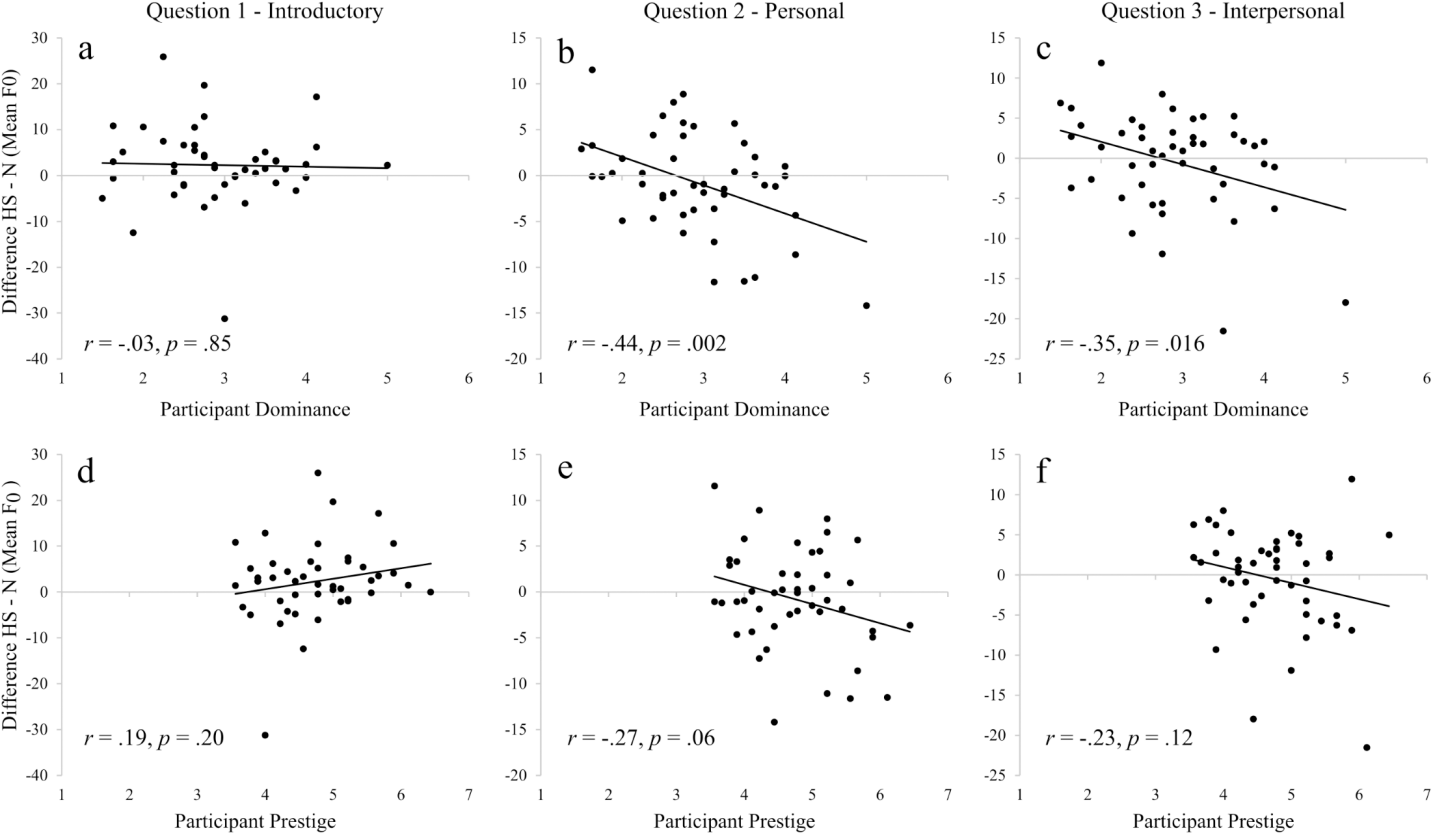
Correlations between participant self-rated status and mean F_0_ difference per question. In all cases, the Y axis represents the difference in F_0_ of responses to High-status (HS) minus Neutral (N) targets, so that a negative value on the Y axis represents a tendency of participants to lower their F_0_ in responses to HS targets (dominant, prestigious), in comparison to the neutral target. For upper panels (a-c), the X axis represents participant PD; in lower panels (d-f), the X axis represents participant PP. Results are split by question: Introductory (left; panels a, d), Personal (centre; panels b, e), and Interpersonal (right; panels c, f).

In general, most differences in vocal parameters were found between the neutral and high-status targets, but not between the two high-status targets; this was particularly true for mean F_0_, in which the interaction between PD and target was significant, and planned contrasts revealed differences in the predicted F_0_ towards the targets, for participants with low and high, but not mean, PD. In fact, the correlation between PD differences in F_0_ towards high-status minus the neutral target–i.e. a difference score (Fig 6), was negative and significant for questions 2 (Personal; Fig 6B) and 3 (Interpersonal; Fig 6C), and only marginally non-significant for question 2, when correlating F_0_ with PP (Fig 6E). This shows that participants who perceive themselves as low in PD tend to speak with higher mean F_0_, or pitch, to high status targets, and conversely participants with high PD tend to lower their mean F_0_.

## Discussion

Previous studies have suggested that manipulations of vocal parameters, particularly F_0_, affect perceived dominance [28], that men adjust their voices during interaction with competitors depending on their perceived relative dominance [25] and, more generally, that hierarchical relationships are dependent on relative, rather than absolute, social status perceptions [33,34]. Such studies have, however, focused on dominance, and predominantly on men’s voices. Our experimental design of a job interview scenario provides new insights into the specific nature of hierarchical relationships and into the vocal differences when addressing dominant and prestigious individuals of both men and women.

Firstly, we found that male and female participants who judged themselves to be more dominant lowered their F_0_ when speaking to all targets, in line with previous research on men [25]. We also found a tendency for more prestigious participants to respond with lower intensity variability, and dominant participants to decrease variability in fundamental frequency (F_0_ SD), which would perhaps make them sound calmer and more in control of situations; in fact, decreased F_0_ variability is associated with lower aggressiveness in industrial as well as foraging societies [48], and it is known to occur in contexts involving competition [7,49].

Differences in vocal parameters between responses to the different targets were strongest in mean F_0_ (Table 2, Table 3), and when including self-perceived dominance in the models. As predicted by previous research [25], participants, and particularly persons who perceive themselves as low in dominance, responded with a relative higher F_0_ when speaking to the high-status targets, the opposite trend to what participants with high dominance tended to do. Additionally, variation in voice modulation arose when comparing responses to the neutral versus the high-status targets, suggesting that the status of the target, whether high in dominance or prestige, is the key factor. In fact, differences between responses to the dominant and prestigious targets were not significantly different when analysing all questions together.

Contextual vocal modulations, however, were not found to occur in mean intensity or intensity SD. This suggests that while these parameters may be a robust cue of social status, as shown above in the effects of self-perceived prestige and intensity variability, or even context-dependent (e.g. shouting) interactions, speakers do not modulate their voice intensity during free speech depending solely on the relative social status of the listeners. This is likely due to the nature of our interview scenario, as participants were not directly competing, and were not trying to signal aggression in front of a potential employer, but rather to make themselves appear favourable for a position.

Furthermore, the use of a job interview scenario allowed us to include questions with different characteristics: introductory, personal, and interpersonal. The analysis of the vocal characteristics by question revealed significant vocal differences dependent on the perceived social status of the target listener, mostly when personal and interpersonal questions are answered, but not during introductory responses. In these cases, the effects of target, especially when including PD as a covariate, were significant (Table 4). In general, participants’ mean F_0_ was raised when responding to the dominant or prestigious targets (Fig 3), and this was stronger in low self-perceived status participants (Fig 4), supporting previous results [25]. However, we also found a tendency of participants who feel high in dominance to lower the their mean F_0_ when responding to the high-status targets The apparent differences between responses to personal and interpersonal questions in comparison to the introductory one may be because each participant tended to introduce him or herself in a similar manner to all targets (e.g. “my name is…”, “I am currently studying…”, “I live in…”), but when confronted with questions that required them to discuss their specific skills to the target (personal), and even more so when asked to imagine a hypothetical interaction with the target (interpersonal), the nature of the questions themselves may have induced participants to *improvise* and respond more naturally.

Differences in vocal parameters between the responses to these questions are apparent in our analysis. Although it could be argued that this is a product of the order in which the questions were presented, we suggest that this is unlikely because of the different characteristics of the questions and, furthermore, because participants participated in three interviews, which meant that they responded to question one (introductory) after question 3 (interpersonal) twice during the experiment. The possibility of order effects could be tested in future experiments, to disentangle responses to different types of questions. In addition, the 28 faces available, from which targets were selected, were all initially constructed under instructions to create a highly prestigious, or highly dominant individual. This could mean that the neutral target, being the median rated face, displayed some level of dominance and/or prestige as opposed to being ‘neither’ dominant nor prestigious. Although more noticeable differences between the targets would likely make differences stronger, our manipulation seems to have been enough to elicit vocal modulations in the participants. Additionally, there is evidence suggesting that men are generally perceived as more dominant than women (see, e.g. [50]), which could be a confounding factor on the issue of dominance for female participants; future studies could address vocal modulations in response to men, but also women, of varying social status. Also, although we created names, personas, and even faces that were high in either prestige or dominance, the prestigious target was perceived as significantly more dominant than the neutral target, and the dominant target as more prestigious than the neutral (Fig 2). This could be because they are both high-status strategies and there is some ambiguity in the literature, and perhaps in real life, about what the differences are between dominance and prestige, or even because are they intrinsically linked, and manoeuvring oneself to high status may require a partly ‘prestigious’ approach and a partly dominant approach, or some combination of the two. We believe that this may be one of the reasons why we see the neutral face as being lower in both, as this person is not likely to attain high status, be it through dominance or prestige. Finally, there is a potentially confounding effect of attractiveness; although the dominant and prestigious targets did differ in perceived attractiveness (S1 Table), it is important to highlight that there were no differences in attractiveness between the neutral and either of the high-status targets (of the two high-status targets, one has lower, and the other has higher, attractiveness that the neutral one). Furthermore, most differences in vocal parameters are found when comparing responses to the neutral versus the two high-status targets (S1 Table and S1 Fig). This problem, however, could be further addressed in future studies, presenting more targets of each social status.

In conclusion, using a novel job interview scenario, we found that self-perceptions of dominance and prestige affected vocal parameters such that the higher an individual’s self-perceived dominance, the lower their mean F_0_, F_0_ SD, and minimum F_0_, and the higher their self-perceived prestige, the lower their intensity variability. Additionally, regardless of self-perceived status, participants changed their vocal characteristics when talking to neutral versus high-status targets, displaying a relatively higher mean F_0_ when talking to high-status targets. The context of questions (i.e. introductory, personal, or interpersonal) also affected participants’ vocal characteristics with the greatest changes in F_0_ according to status of the listener observed for the responses to the personal and interpersonal questions. These F_0_ effects were most pronounced when including participant self-perceived dominance in the models. Ultimately our findings suggest that individuals’ vocal characteristics are influenced, whether consciously or non-consciously, by the relative difference between their self-perceived social status and the social status of the listeners.

## Supporting information

S1 Fig Ratings of targets’ attributes given by the participants. Results are split by target (neutral: white bars; dominant: light grey bars; prestigious: dark grey bars) and attribute rated. a) Facial images; b) Employee testimonials; c) Names; d) Job titles. Bars represent mean ± 1 s.e.m.
(TIF)

S1 Table Independent ratings of target attributes. Mean ratings for each attribute (images, employee testimonials, names, and job titles). N = Neutral target, D = Dominant target, P = Prestigious target. Results are from repeated-measures general linear models (d.f. = 2, 42 in each case) for each rated attribute. Significant effects are in bold.
(XLSX)

S2 Table Descriptive statistics for all vocal parameters. Results represent mean ± SD for male and female participants to each type of target (neutral, dominant, prestigious).
(XLSX)

S3 Table Context-dependent variation in vocal parameters. T = Target (neutral, dominant, prestigious), Q = Question, PD = Participant Dominance, PP = Participant Prestige, PS = Participant Sex (male, female). Results are from repeated-measures general linear models for each vocal parameter, with Holm–Bonferroni adjustment for multiple tests. Significant effects are in bold. *Sphericity could not be assumed and Greenhouse–Geisser correction was used. Interactions with a covariate (PD, PP) and the main effect of those covariates, are taken from the ANCOVA. All other effects are taken from an ANOVA [see, 41] on the same data without the covariate.
(XLSX)

S4 Table Planned contrasts estimated for participants with varying degrees of dominance and prestige. T = Target (neutral, dominant, prestigious), Q = Question, PS = Participant Sex, N = Neutral Target, HS = High-status Targets (dominant, prestigious), D = Dominant Target, P = Prestigious Target. For participants, self-perceived status covariates (PD = Participant Dominance, PP = Participant Prestige) were centred to low (10th percentile), mean, and high (90% percentile) levels. Results are from planned contrasts (Helmert) for each vocal parameter (d.f. = 1, 41), including only PD or PP as a covariate. All values are taken from an ANCOVA. Significant effects are in bold. Of particular interest, is the main effect of target (T), and its changes for participants with different levels of dominance or prestige.
(XLSX)

S5 Table Planned contrasts estimated for participants with varying degrees of dominance and prestige, per question. T = Target (neutral, dominant, prestigious), N = Neutral Target, HS = High-status Targets (dominant, prestigious), D = Dominant Target, P = Prestigious Target. For participants, self-perceived status covariates (PD = Participant Dominance, PP = Participant Prestige) were centred to low (10th percentile), mean, and high (90% percentile) levels. Results are from planned contrasts (Helmert) for each vocal parameter (d.f. = 1, 43; 1, 44; and 1, 45 for question 1, 2, and 3 respectively), including only PD or PP as covariates. All values are taken from an ANCOVA. Significant effects are in bold. Of particular interest, is the main effect of target (T), and its changes for participants with different levels of dominance or prestige.
(XLSX)

S1 Text Supplementary materials and methods. (DOCX)

S1 Data Excel file with data on participants and analysed acoustic characteristics. (XLSX)
