# Supplementary figures and images for: Perceived differences in social status between speaker and listener affect the speaker's vocal characteristics
Source: PLoS One. 2017 Jun 14;12(6):e0179407. doi: 10.1371/journal.pone.0179407 (PMC5470693; doi:10.1371/journal.pone.0179407)

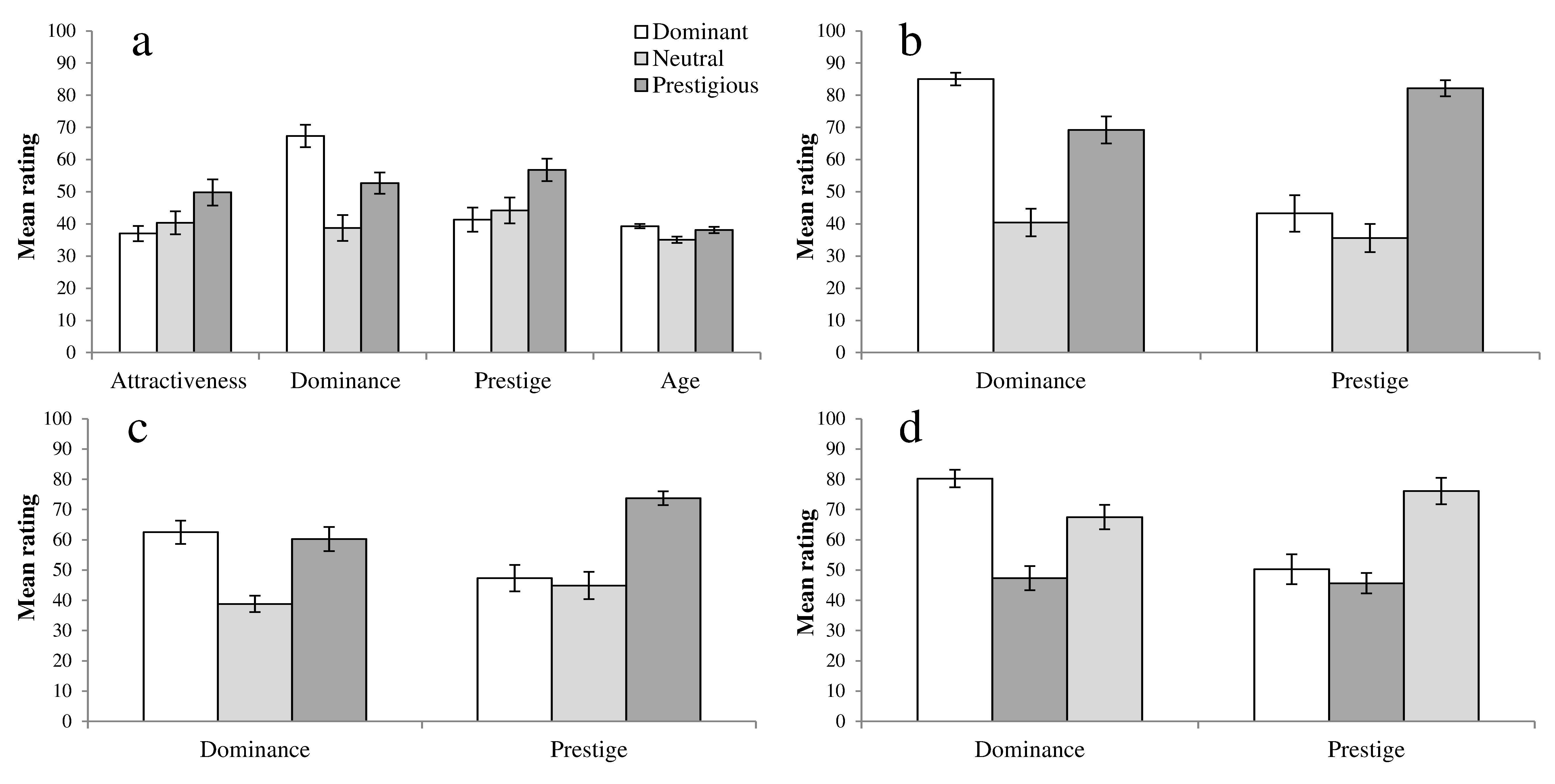

Supplement: S1 Fig — Results are split by target (neutral: white bars; dominant: light grey bars; prestigious: dark grey bars) and attribute rated. a) Facial images; b) Employee testimonials; c) Names; d) Job titles. Bars represent mean ± 1 s.e.m. (TIF) [file pone.0179407.s001.tif]
