# Supplementary material for: Perceived differences in social status between speaker and listener affect the speaker's vocal characteristics
Source: PLoS One. 2017 Jun 14;12(6):e0179407. doi: 10.1371/journal.pone.0179407 (PMC5470693; doi:10.1371/journal.pone.0179407)
Supplement: S1 Text — (DOCX) [file pone.0179407.s007.docx]

**S1 Text. Supplementary Materials and Methods**

**Target** **stimuli**

21 raters (14 men, mean age ± SD = 30.7 ± 9.6; 7 women, 35.4 ± 10.1) independently judged each attribute of the targets (names, job titles, testimonials, and faces).

**Experimental procedure**

At the end of the experiment, participants were asked to write what they thought the purpose of the study was. 39.6% of the participants (7 men, 12 women) had a relatively accurate idea about our manipulation (i.e. they understood we were manipulating the dominance and prestige of the targets). However, it is important to highlight that because this was the last part of the experiment, participants responded to this question after rating all the targets for both dominance and prestige (which should have given them a good idea of what the real purpose of the experiment was). No participant, however, realised that the main focus of the study was to perform an acoustic analysis of their voices.
